# Ferroptosis-Related Gene *MT1G* as a Novel Biomarker Correlated With Prognosis and Immune Infiltration in Colorectal Cancer

**DOI:** 10.3389/fcell.2022.881447

**Published:** 2022-04-20

**Authors:** Bi Peng, Jinwu Peng, Fanhua Kang, Wenqin Zhang, Emin Peng, Qingchun He

**Affiliations:** ^1^ Department of Pathology, Xiangya Hospital, Central South University, Changsha, China; ^2^ Department of Pathology, Xiangya Changde Hospital, Changde, China; ^3^ Xiangya International Medical Center, Xiangya Hospital, Central South University, Changsha, China; ^4^ National Clinical Research Center for Geriatric Disorders, Xiangya Hospital, Central South University, Changsha, China; ^5^ Department of Emergency, Xiangya Hospital, Central South University, Changsha, China; ^6^ Department of Emergency, Xiangya Changde Hospital, Changde, China

**Keywords:** colorectal cancer, ferroptosis, MT1G, prognosis, immune response

## Abstract

Ferroptosis, a newly discovered way of cell death, has been proved to be involved in the oncogenesis and development of cancers, including colorectal cancer (CRC). Here, by identifying the differentially expressed genes (DEGs) from three CRC transcriptome microarray datasets (GSE20842, GSE23878, and GSE25070), we found that the expression of MT1G was significantly decreased in CRC tissues, and the patients with a high level of MT1G displayed a poor prognosis. Quantitative PCR (qPCR) further confirmed the downregulated MT1G in two CRC cells, HCT8 and HCT116. The colony-forming assay indicated that the MT1G overexpression exhibited a remarkable inhibition of cell proliferation in HCT8 and HCT116 cells. In addition, we explored the co-expressed genes of MT1G to gain a better understanding of its potential signaling pathways. Aberrantly expressed MT1G also affected the immune response of CRC patients. Collectively, these findings might deepen our comprehension on the potential biological implications of MT1G in CRC.

## Introduction

Colorectal cancer (CRC) is one of the most common malignant tumors in humans, with the fourth highest mortality among cancer-related diseases (Yang et al., 2019; Deng et al., 2021). There has been significant progress in the development of diagnosis and treatment in CRC patients (Johdi and Sukor, 2020), whereas the therapeutic effect of CRC is still not satisfactory. Therefore, it is essential to explore and provide a novel targeted agent for an update on prognosis and therapeutics of colorectal cancer patients.

Ferroptosis is a common non-apoptotic form of cell death, which is caused by acute or chronic cellular stress due to abnormal metabolic and biochemical processes (Yan et al., 2021). Increasing evidence has demonstrated the roles of ferroptosis-related genes in tumorigenesis, treatment, and immune regulation in cancers, such as CRC, hepatocellular carcinoma, breast cancer, and so on (Li et al., 2021; Xu et al., 2021; Zhang et al., 2021). For instance, *β*-elemene, a new ferroptosis inducer, acts as a cetuximab sensitizer in KRAS-mutated CRC cells (Chen et al., 2020). F-box and WD repeat domain-containing 7 (FBW7) have the ability to activate ferroptosis to strengthen the toxicity of gemcitabine-targeting pancreatic adenocarcinoma cells (Ye et al., 2021). Talaroconvolutin A (TalaA) could display the cancer inhibitory activity by inducing cell ferroptosis (Xia et al., 2020).

Metallothionein-1G (MG1T), a ferroptosis-related gene, is a member of metallothioneins (MTs) involved in the regulation of oxidation and metalloproteinases (Si and Lang, 2018). To date, several studies have indicated the potential roles of MG1T in cancers. For example, Sun et al. have demonstrated that abnormally expressed MG1T facilitates the resistance of hepatocellular carcinoma cells to sorafenib by inhibiting ferroptosis (Sun et al., 2016). Likewise, MG1T has also been reported to be involved in ferroptosis of esophageal adenocarcinoma (Zhu et al., 2021). However, the specific mechanism of MT1G in CRC has not been elucidated.

Here, we used multiple public databases to investigate the functional roles of MT1G in the progression and prognosis of patients with CRC. The relevant signaling pathways and biological functions of MT1G were also analyzed systematically. Then, we also evaluated that aberrantly expressed MT1G might affect the immune response of CRC patients. Thus, our studies could provide a new perspective to understand the important roles of MT1G in the progression and treatment of CRC.

## Materials and Methods

### Data Acquisition

The CRC datasets (GSE20842 (Gaedcke et al., 2010), GSE23878 (Uddin et al., 2011), and GSE25070 (Hinoue et al., 2012)) were obtained from the GEO database, which provides an invaluable resource of publicly available gene expression data and other functional genomics data (Clough and Barrett, 2016). We established the differentially expressed genes (DEGs) according to the following criteria: *p*-value < 0.05 and | logFC| ≥ 1.5. Venn analysis was performed by FunRich to search for co-DEGs between the three GEO datasets and a ferroptosis-related gene dataset (Zhou and Bao, 2020).

### Expression Profile Analysis

Transcriptome data of 647 CRC cases and 51 adjacent tumor samples were collected and analyzed from TCGA database (McCain, 2006). Furthermore, multiple databases are used together to validate the MT1G expression, such as GEPIA2 (Tang et al., 2019), UALCAN (Chandrashekar et al., 2022), and TNMplot (Bartha and Győrffy, 2021).

### Correlation Analysis

We used the LinkedOmics database (Vasaikar et al., 2018) to obtain the co-expressed genes associated with MT1G. These results were visualized *via* a volcano plot and heatmaps. In addition, Gene Set Enrichment Analysis (GSEA) and Kyoto Encyclopedia of Genes and Genomes (KEGG) pathways were also conducted by LinkedOmics to reveal the potential biological functions of MT1G.

### Immunological Analysis

The single-sample GSEA (ssGSEA) algorithm was exploited to evaluate the correlations between the MT1G expression and tumor-infiltrating immune cells. Subsequently, these associations were validated using TIMER2.0 (Li et al., 2020) and TISIDB (Ru et al., 2019). We also utilized TISIDB to get an evaluation of MT1G in the regulations of immunomodulators and chemokines.

### Cell Lines and Culture Conditions

CRC cell lines (HCT8 and HCT116) and human colorectal epithelium NCM460 cell lines were acquired from the Center for Molecular Medicine, Xiangya Hospital. These cell lines were cultured in Dulbecco’s Modified Eagle’s Medium (Gibco, United States), which contained 10% FBS (Gibco, United States) and 100 µ/ml streptomycin/penicillin (Gibco, United States), and were incubated at 37°C in a humidified incubator with 5% CO_2_.

### Quantitative PCR

We used TRIzol reagent (Invitrogen, United States) to extract total RNA from different cell lines. Then, total RNA was reverse transcribed into cDNA using the PrimeScriptTM strand cDNA synthesis kit (Takara, China). Quantitative PCR (qPCR) was conducted to assess the expression level of MT1G. The primers of MT1G were 5′-GAC​CCC​AAC​TGC​TCC​TGT​G-3′ and 5′-ACT​TCT​CCG​ATG​CCC​CTT​TG-3′. *β*-actin served as a quantitative gene for internal control.

### Colony Forming Assay

We utilized MT1G-Flag-pcDNA3.1 to transfect the HCT8 and HCT116 cells for 48 h. Then, about 1,000 cells were implanted in six-well dishes and cultured for about 2 weeks. The visible colonies were counted by crystal violet staining.

### Statistical Analysis

SPSS 19.0 software was utilized to make a statistical analysis. All experimental results were shown as mean ± standard deviation (SD). Student’s t-test was exploited to analyze the expression of MT1G in tumor and normal tissues. A *p*-value of less than 0.05 was considered statistically significant.

## Results

### Identification of the Downregulated Ferroptosis-Related Gene *MT1G* in CRC Tissues and Cells

**FIGURE 1:**
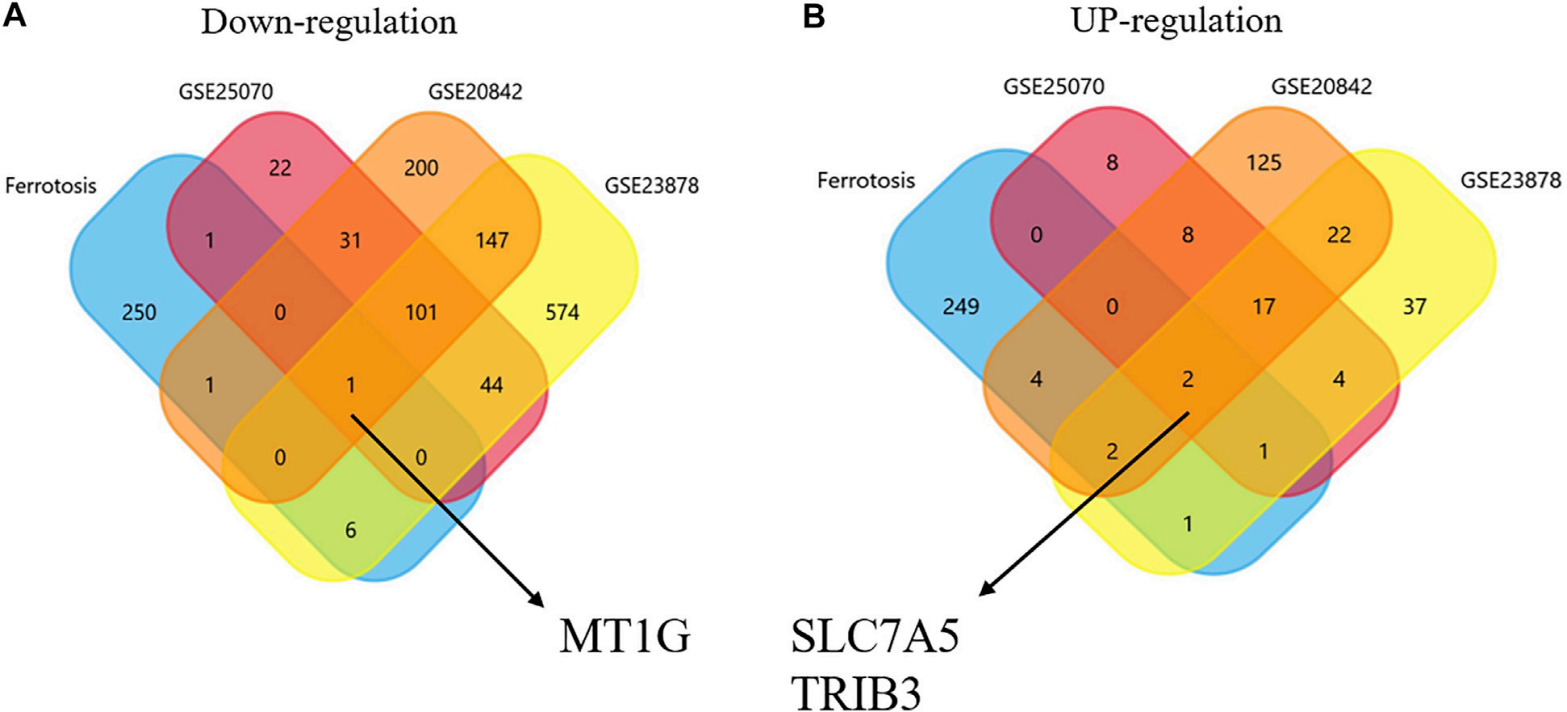
Identification of ferroptosis-associated genes in CRC. **(A)** Downregulated co-DEGs identified by Venn diagrams. **(B)** Upregulated co-DEGs identified by Venn diagrams.

**FIGURE 2:**
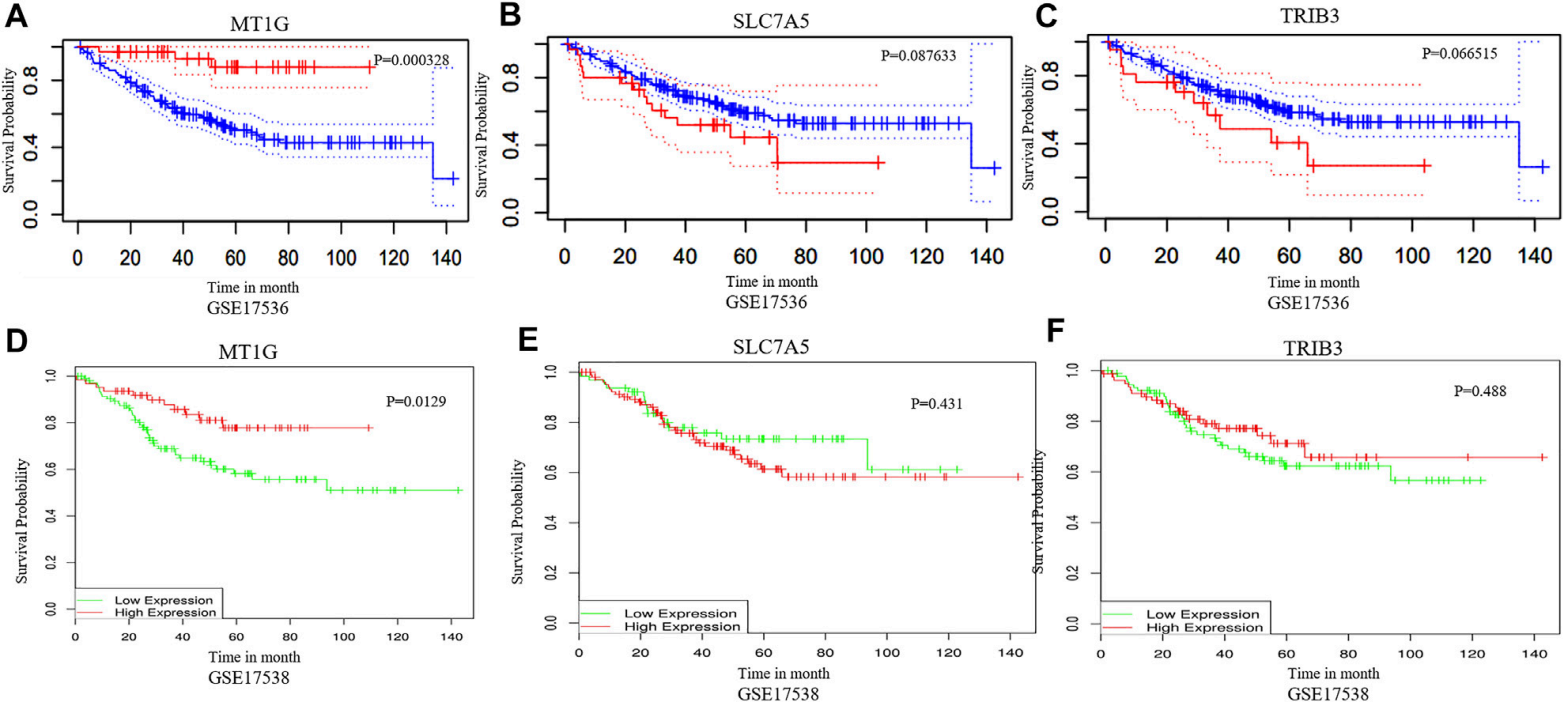
Prognostic value analysis of MT1G, SLC7A5, and TRIB3 in CRC. **(A–F)** PrognoScan database and DRUGSURV database indicated the prognostic values of MT1G, SLC7A5, and TRIB3 in CRC patients.

The DEGs in CRC and normal tissues were extracted from three GEO datasets (Supplementary Table S1). Venn plots were obtained from among these three GEO datasets and the ferroptosis-related gene dataset. Figure 1A shows one co-downregulated gene (*MT1G*), and Figure 1B shows two co-upregulated genes (*SLC7A5* and *TRIB3*) (Figures 1A,B). Then, the PrognoScan database (Mizuno et al., 2009) and DRUGSURV database (Amelio et al., 2014) were used to explore the prognostic values of these ferroptosis-related genes in CRC patients from GSE17536 and GSE17538. As shown in Figures 2A–F, a high level of *MT1G* indicated good prognosis in patients with CRC. However, *SLC7A5* and *TRIB3* have no significant effects on the prognostic values.

**FIGURE 3:**
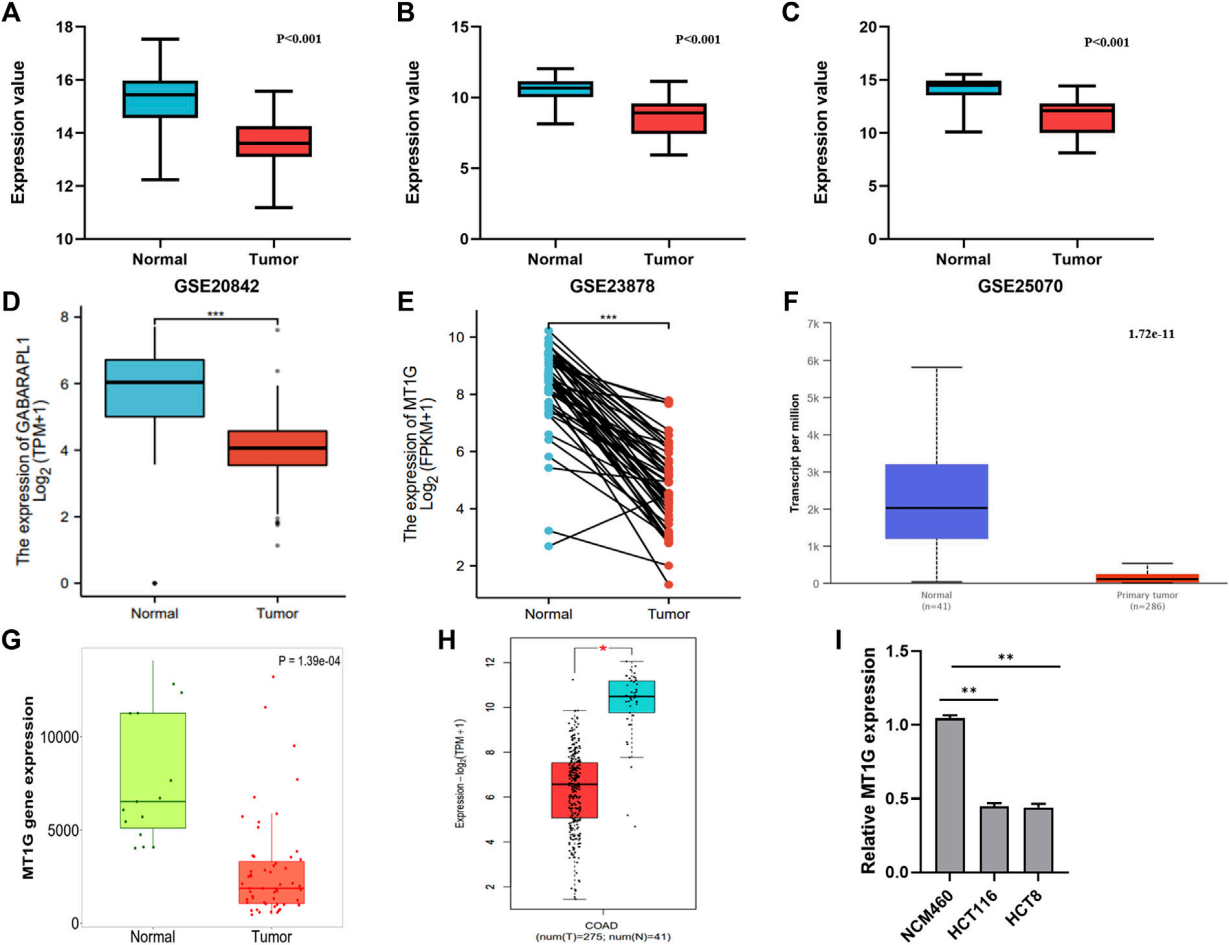
Expression levels of MT1G verified in CRC tissues and cells. **(A–H)** Comparison of the MT1G expression between CRC and normal controls from **(A)** GSE20842, **(B)** GSE23878, **(C)** GSE25070, **(D)** paired TCGA-CRC, **(E)** unpaired TCGA-CRC, **(F)** UALCAN, **(G)** TNMplot, and **(H)** GEPIA2. **(I)** Low expression of MT1G was detected by qPCR. *p < 0.05; **p < 0.01; and ***p < 0.001.

**FIGURE 4:**
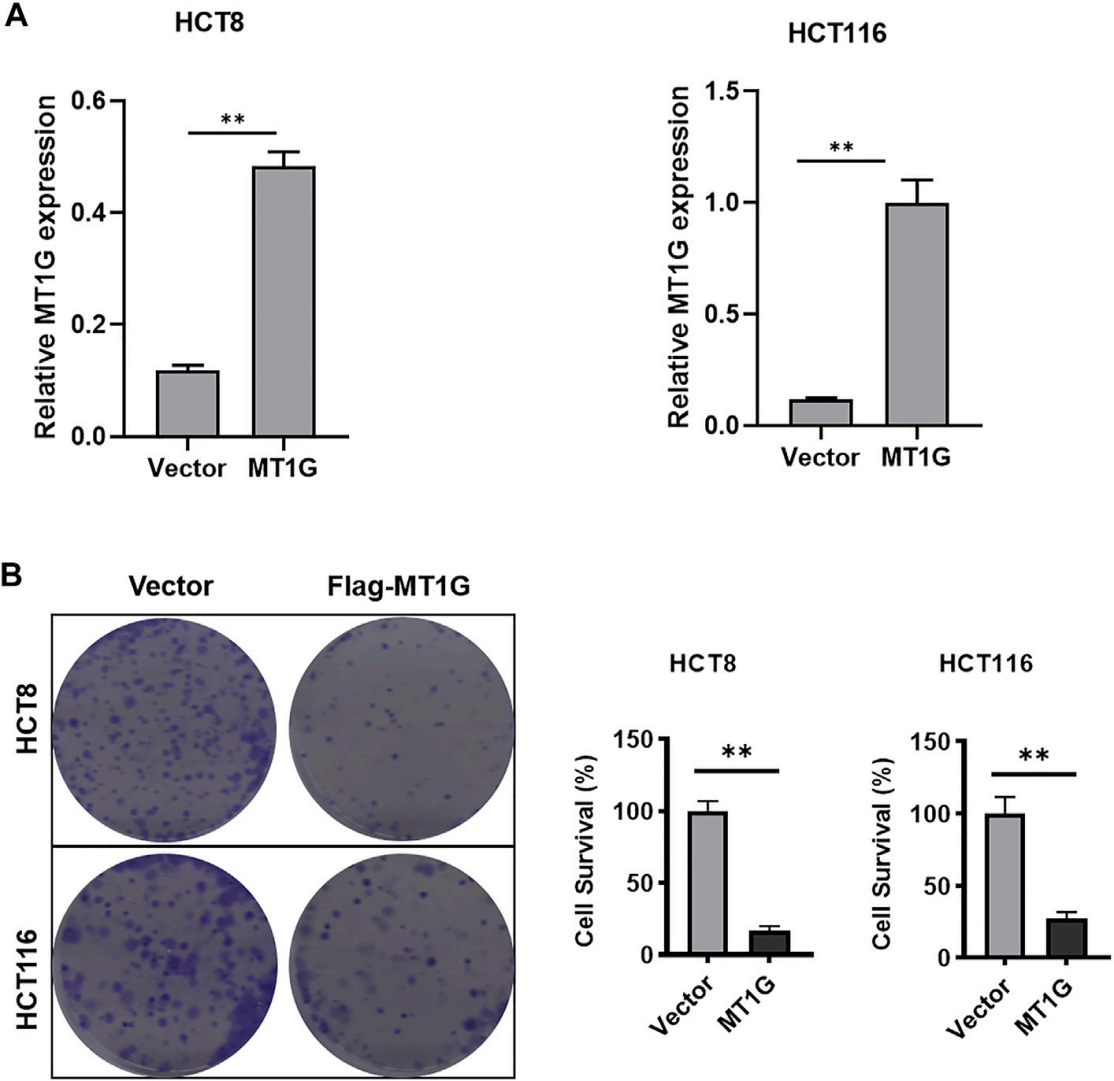
Overexpression of MT1G inhibited CRC cell growth. **(A)** Overexpression of MT1G in HCT8 and HCT116 cells. **(B)** Colony formation rate of HCT8 and HCT116 cells after the MT1G overexpression. *p < 0.05; **p < 0.01; and ***p < 0.001.

In addition, the downregulated MT1G was confirmed in CRC patients from GSE20842 (Figure 3A), GSE23878 (Figure 3B), and GSE25070 (Figure 3C). Our works on TCGA-CRC also suggested the low MT1G expression in both unpaired (Figure 3D) and paired tissues (Figure 3E). Moreover, all of the other databases, including UALCAN (Figure 3F), TNMplot (Figure 3G), and GEPIA (Figure 3H), demonstrated that the expression levels of *MT1G* were downregulated in CRC. The reduced *MT1G* expression was further confirmed in CRC cells, HCT8 and HCT116, using qPCR (Figure 3 I). In addition, the overexpression of *MT1G* in HCT116 and HCT8 significantly inhibited the colony formation rate (Figures 4A,B). Thus, these data indicated the important roles of downregulated MT1G in CRC.

### Co-expression Analysis of MT1G in CRC

**FIGURE 5:**
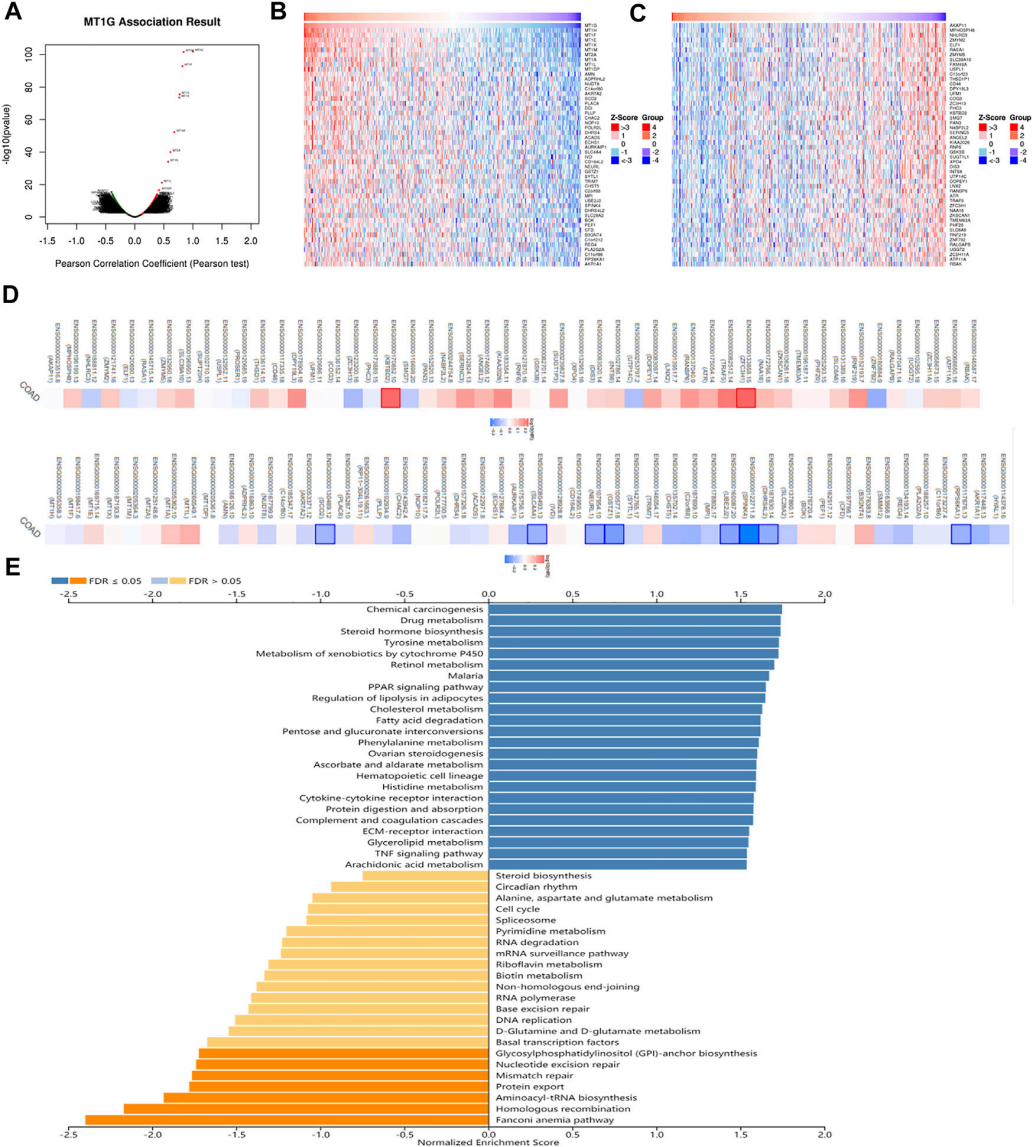
Co-expression analysis of MT1G in CRC. **(A)** Genes remarkably related to MT1G by Pearson’s test. **(B,C)** Heatmap indicated the top 50 positively and negatively co-expressed genes of MT1G. **(D)** Survival map of the top 50 positively or negatively co-expressed genes of MT1G. **(E)** GSEA analyses indicated the potential signaling pathways regulated by MT1G.

To investigate the biological roles of MT1G in CRC, we used LinkedOmics to obtain the co-expression analysis of MT1G. Figure 5A indicated that there were 3954 genes positively associated with MT1G and 3337 genes negatively associated with MT1G. The top 50 genes having a positive or negative correlation with MT1G are shown in Figures 5B,C. Moreover, these genes had an influence on the prognosis of patients with CRC. Out of 50 positive genes, 29 owned a protective hazard ratio (HR), indicating their favorable roles on the prognosis. Conversely, 32 out of 50 negative genes owned adverse HR, indicating their unfavorable roles on the prognosis (Figure 5D). Furthermore, GSEA was designed to evaluate the underlying biological function of MT1G in CRC. As shown in Figure 5E, the top three signaling pathways were the Fanconi anemia (FA) pathway, homologous recombination, and aminoacyl-tRNA biosynthesis.

### Roles of MT1G in the Immune Microenvironment

**FIGURE 6:**
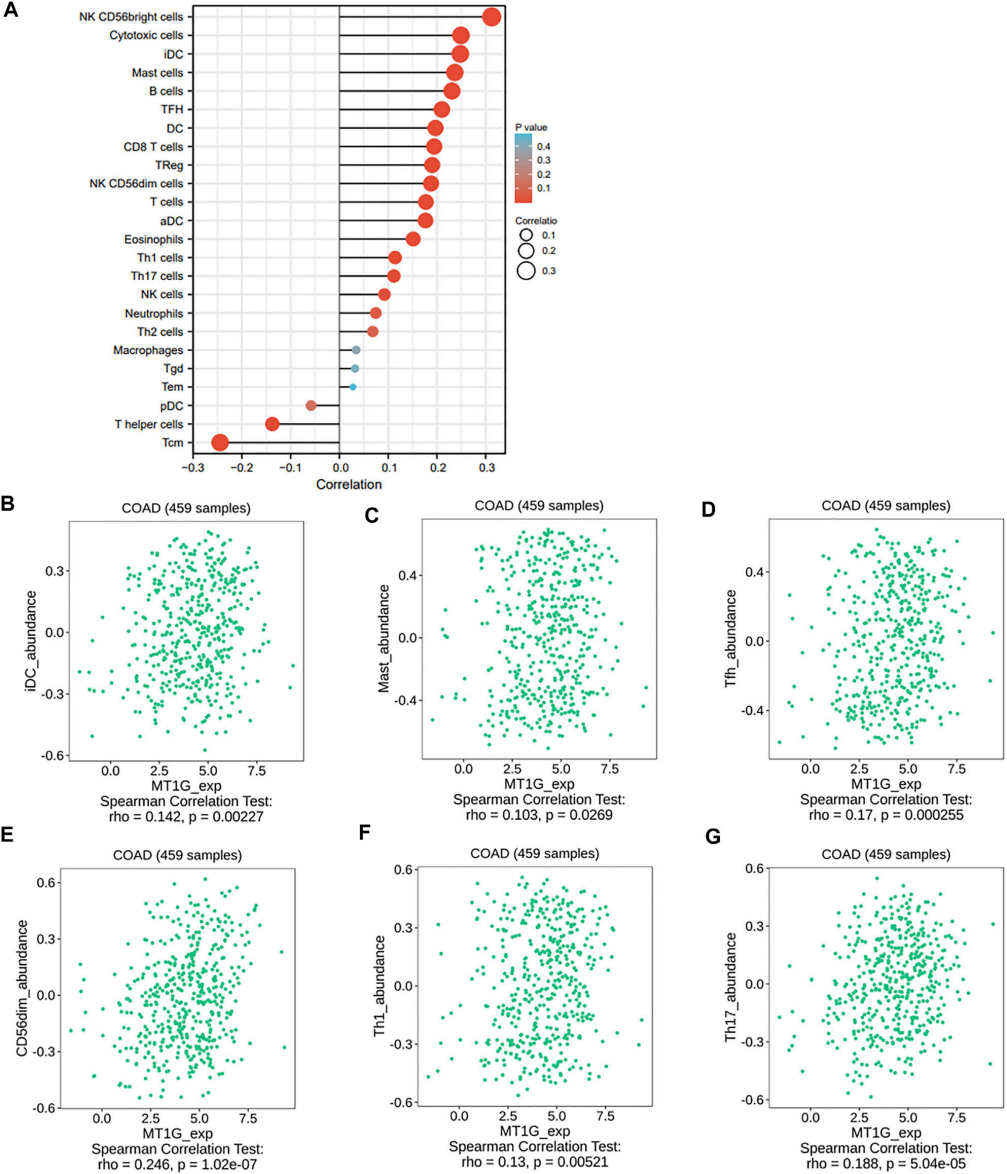
Relationship of immune infiltration cells and the MT1G expression. **(A)** Correlations between a variety of immune infiltration cells and MT1G expression though the ssGESA algorithm. **(B–G)** Associations between MT1G with several immune cells, such as iDCs, mast cells, NK CD56dim cells, Th1 cells, and Th17 cells, were further validated by the TISIDB database.

**FIGURE 7:**
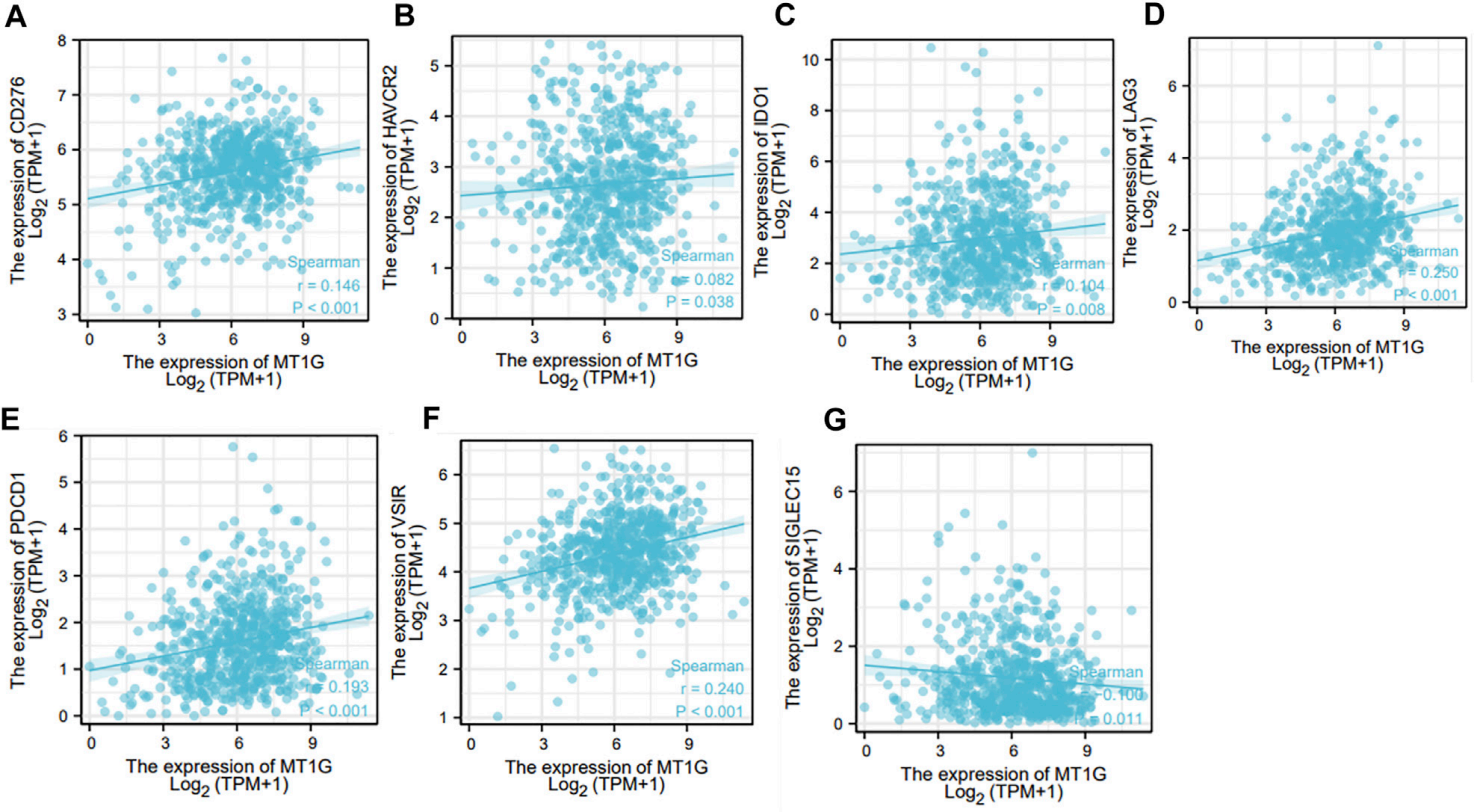
Association between MT1G and several immune checkpoints. **(A–G)** Several immune checkpoints, such as **(A)** CD267, **(B)** HAVCR2, **(C)** IDO1, **(D)** LAG3, **(E)** PDCD1, **(F)** VSIR, and **(G)** SIGLEC15, displayed significant association with the MT1G expression in CRC.

Given the important roles of ferroptosis regulators in the immune response (Friedmann Angeli et al., 2019), we used the ssGSEA algorithm to analyze the association between the ferroptosis-associated gene *MT1G* and immune infiltration cells. As shown in Figure 6A, several immune cells, such as immature dendritic cells (iDCs), mast cells, TFH cells, NK CD56dim cells, Th1 cells, and Th17 cells, were all positively associated with MT1G, and the TISIDB database was used to confirm these associations (Figures 6B–G). We also investigated the associations between the MT1G level and several immune checkpoints. Figures 7A–F indicate the positive correlations between the expression of MT1G and multiple immune checkpoints, such as B7 homolog 3 protein (CD276), hepatitis A virus cellular receptor 2 (HAVCR2), indoleamine 2,3-dioxygenase 1 (IDO1), lymphocyte-activation gene 3 (LAG3), programmed cell death 1 (PDCD1), and V-Set immunoregulatory receptor (VSIR). Figure 7G implies the negative association between the MT1G expression level and sialic acid-binding Ig-like lectin 15 (SIGLEC15).

The following works focused on the other immunoregulation effects of MT1G in CRC. The TISIDB database was exploited to verify the roles of MT1G in several immune signatures, including immunostimulators, immunoinhibitors, chemokines, and chemokine receptors. Supplementary Figure S1A shows that the tumor necrosis factor receptor superfamily member 18 (TNFRSF18) was the most significant immune-stimulating molecule, followed by tumor necrosis factor receptor superfamily member 13C (TNFRSF13C), tumor necrosis factor ligand superfamily member 9 (TNFSF9), and tumor necrosis factor receptor superfamily member 7 (CD27). Supplementary Figure S1B displays the top four immunosuppressive molecules containing LAG3, gamma-glutamyltransferase 1 (CD224), PDCD1, and kinase insert domain-containing receptor (KDR). Meanwhile, the associations between MT1G and chemokines and receptors were explored. As shown in Supplementary Figure S2A, C-C chemokine ligand 5 (CCL5), C-C chemokine ligand 2 (CCL2), C-C chemokine ligand 23 (CCL23), and C-C chemokine ligand 28 (CCL28) were the most significantly positive chemokines. Moreover, C-C chemokine receptor type 10 (CCR10), chemokine receptor 5 (CXCR5), chemokine receptor 6 (CXCR6), and C-C chemokine receptor type 7 (CCR7) were the most significantly positive receptors (Supplementary Figure S2B). Taken together, these findings showed the underlying function of MT1G in the immune regulation in CRC patients.

## Discussion

Our team’s work identified that the ferroptosis-associated gene, *MT1G*, had a great impact on the development and progression of CRC. Its high expression implied a good prognosis. In addition, our findings have also suggested that MT1G was significantly correlated with the immune microenvironment of CRC patients. It might play an important role in the prognosis of CRC patients by regulating the immune response. In general, these results have indicated that MT1G has a great capacity for being considered a therapeutic biomarker for patients with CRC.

Ferroptosis, a newly discovered way of iron-dependent oxidative cell death, was realized though the regulation of lipid peroxidation (Wang et al., 2021a; Chen et al., 2021). An increasing body of evidence has suggested the functional roles of ferroptosis in CRC tumorigenesis and treatment (Tang et al., 2021). The activation of hypoxia-inducible factor 2α (HIF-2α) is one of the ferroptosis-associated features, potentiating oxidative cell death in CRC cells (Singhal et al., 2021). Lipocalin 2 participates in the regulation of iron homeostasis. It can suppress ferroptosis and facilitate the therapeutic resistance of CRC cells (Chaudhary et al., 2021). Moreover, several signaling pathways are also involved in the regulation of ferroptosis. Ferroptosis can be induced by inhibiting the KIF20A/NUAK1/PP1β/GPX4 signaling pathway in CRC cells, thus improving the oxaliplatin sensitivity (Yang et al., 2021). In our research, the expression of MT1G showed an obvious decrease in CRC tissues and cells. The MT1G overexpression inhibited the cell growth and has an effect on immune regulation. These results suggested that MT1G might be a potential biomarker for CRC patients.

GSEA assessment results indicated that the FA pathway, homologous recombination, and aminoacyl-tRNA biosynthesis were the top three signal pathways in which MT1G-associated genes might be involved. FA is a genetic disorder participating in tumorigenesis and development (Nalepa and Clapp, 2018). Studies have demonstrated the vital roles of the FA-associated DNA damage repair pathway in the inherited predisposition to CRC (Esteban-Jurado et al., 2016). Homologous recombination deficiency (HRD) is one of the common drivers for tumorigenesis (Nguyen et al., 2020). The cancer cells with HRD frequently display high sensitivity to platinum chemotherapy drugs and poly(ADP-ribose) polymerase inhibitors in several malignancies, including CRC (Papageorgiou et al., 2021; Moretto et al., 2022). Aminoacyl-tRNA biosynthesis requires the activity of aminoacyl-tRNA synthetases. Abnormal signaling pathways modulated by aminoacyl-tRNA synthetases exhibit a promising effect on the development of CRC (Zhou et al., 2020). These findings further suggested the biological functions of MT1G in CRC.

The immune microenvironment consists of a variety of immune cells and is widely considered a useful predictor for treatment outcomes, particularly immunotherapy (Hinshaw and Shevde, 2019). Cancer immunotherapy has dramatically changed the strategies for cancer patients by targeting the immune system (Wang et al., 2021b). CRC has a high incidence and poor prognosis worldwide. It is essential to explore novel biomarkers and therapies for improving and prolonging the life of patients with CRC. Nowadays, immunotherapy has rapidly become the mainstay of treatment for CRC. Pembrolizumab and nivolumab, the primary PDCD1 inhibitors, have gained the Food and Drug Administration (FDA) approval for the CRC patients with high microsatellite instability (MSI-H) (Ganesh et al., 2019). Our study also identified the potential roles of MT1G in the immune microenvironment in CRC. Several immune cells, such as iDCs, mast cells, NK CD56dim cells, Th1 cells, and Th17 cells, have significant positive correlations with MT1G. Furthermore, several immunostimulators (TNFRSF18, TNFRSF13C, TNFSF9, and CD27), immunoinhibitors (LAG3, CD224, PDCD1, and KDR), chemokines (CCL5, CCL2, CCL23, and CCL28), and chemokine receptors (CCR10, CXCR5, CXCR6, and CCR7) were also found to be closely related to MT1G. Mast cells are regarded as important regulators for shaping the tumor microenvironment (TME). After entering the TME, mature mast cells could produce angiogenic mediators and growth factors that ultimately promote accelerated cancer cell growth (Derakhshani et al., 2019). TNFSF9 was mainly expressed in tumor-associated macrophages and upregulated in the CRC microenvironment (Wu et al., 2021). High macrophage infiltration has been reported to be associated with improved survival in CRC patients (Forssell et al., 2007). PDCD1, which acts as an immune checkpoint, usually had a higher expression to generate an immunosuppressive TME (Patel and Kurzrock, 2015). However, PDCD1 immune checkpoint inhibitors have been approved to treat metastatic CRC (Gelsomino et al., 2016; Zengin et al., 2021). CXCR5 is the receptor of CXCR13, acting as an inflammatory factor. It has been reported that the CXCL13-CXCR5 axis might activate the PI3K/AKT pathway to enhance the growth and metastasis of CRC cells (Zhu et al., 2015). In our study, aberrantly expressed MT1G might have the regulatory effects on multiple immune signaling pathways in CRC, implying that MT1G could be developed as a novel target of immunotherapy.

There were several limitations to this study. These retrospective data were mainly obtained from several open-access databases, and we have only confirmed the downregulated MT1G in CRC cells using the qPCR method. Future research should focus on the exploration of underlying mechanisms and clinical values of MT1G in CRC.

## Conclusion

In summary, this study illustrated the downregulated ferroptosis-associated gene *MT1G* and revealed that a low MT1G level displayed a worse prognosis in CRC patients. In addition, the MT1G expression was closely related to the immune microenvironment and involved in the progression of tumors. Therefore, *MT1G*, the ferroptosis-related gene, could serve as a potential prognostic biomarker and immune-regulation factor for CRC.

## Data Availability

The datasets presented in this study can be found in online repositories. The names of the repository/repositories and accession number(s) can be found in the article/Supplementary Material.
