# Supplementary material for: Ferroptosis-Related Gene MT1G as a Novel Biomarker Correlated With Prognosis and Immune Infiltration in Colorectal Cancer
Source: Front Cell Dev Biol. 2022 Apr 20;10:881447. doi: 10.3389/fcell.2022.881447 (PMC9065264; doi:10.3389/fcell.2022.881447)
Supplement: Supplementary file 1 [file Table1.DOCX]

Table 1. The mainly characteristic gene expression profiles of three transcriptome microarray GEO datasets.

| GEO datasets | Platform | Sample size | DEGs | Co-DEGs | References |
| --- | --- | --- | --- | --- | --- |
|  |  | Cancer Normal |  |  |  |
| GSE20842 | GPL4133 | 65 65 | 180 up-regulated genes and  481 down-regulated genes | 19 up-regulated genes and  102 down-regulated genes | 20725992 |
| GSE23878 | GPL570 | 35 24 | 86 up-regulated genes and  873 down-regulated genes |  | 21281787 |
| GSE25070 | GPL6883 | 26 26 | 40 up-regulated genes and  200 down-regulated genes |  | 20027224 |

l
