# Supplementary figures and images for: Ferroptosis-Related Gene MT1G as a Novel Biomarker Correlated With Prognosis and Immune Infiltration in Colorectal Cancer
Source: Front Cell Dev Biol. 2022 Apr 20;10:881447. doi: 10.3389/fcell.2022.881447 (PMC9065264; doi:10.3389/fcell.2022.881447)

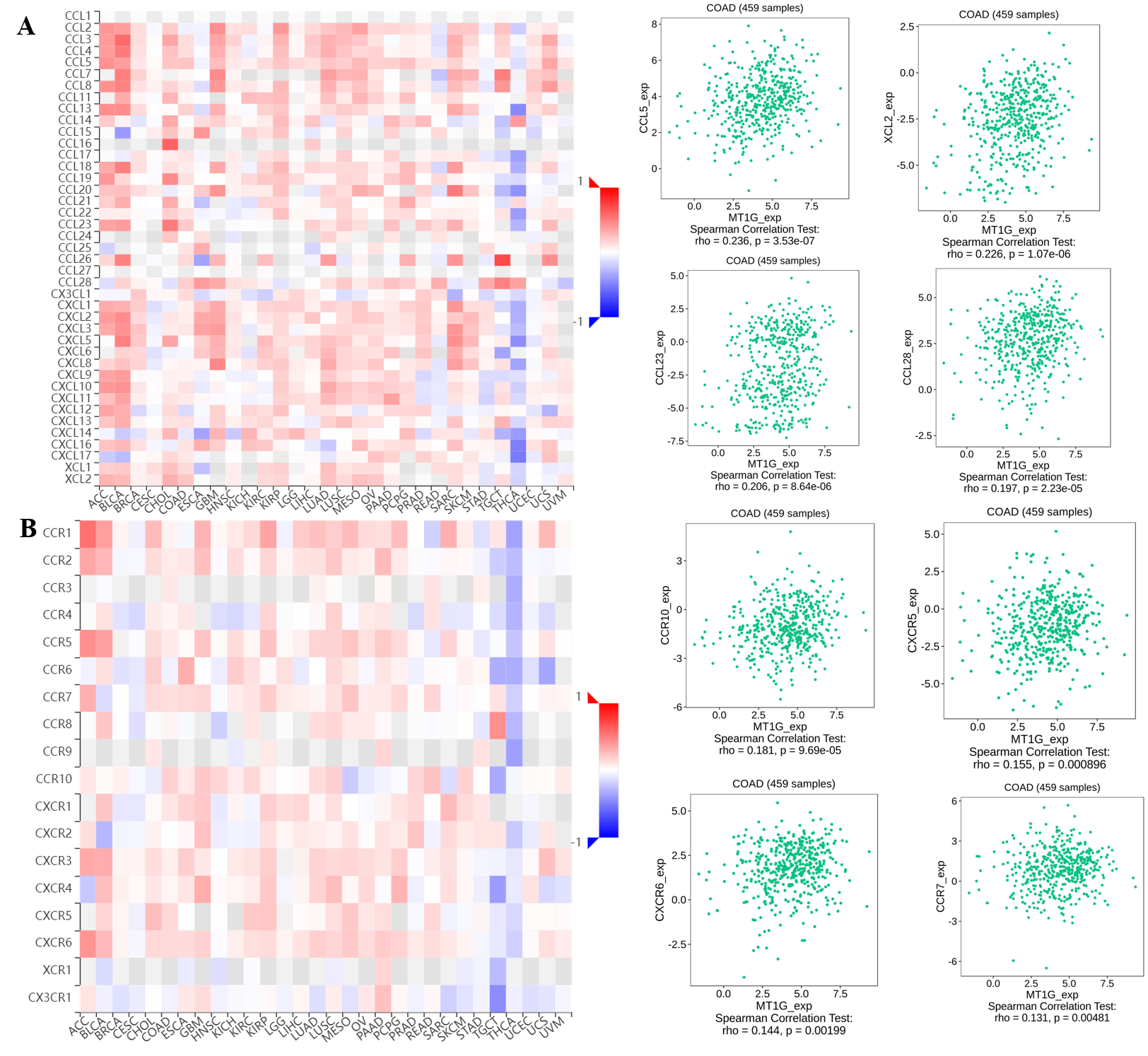

Supplement: Supplementary file 2 [file Image2.TIF]

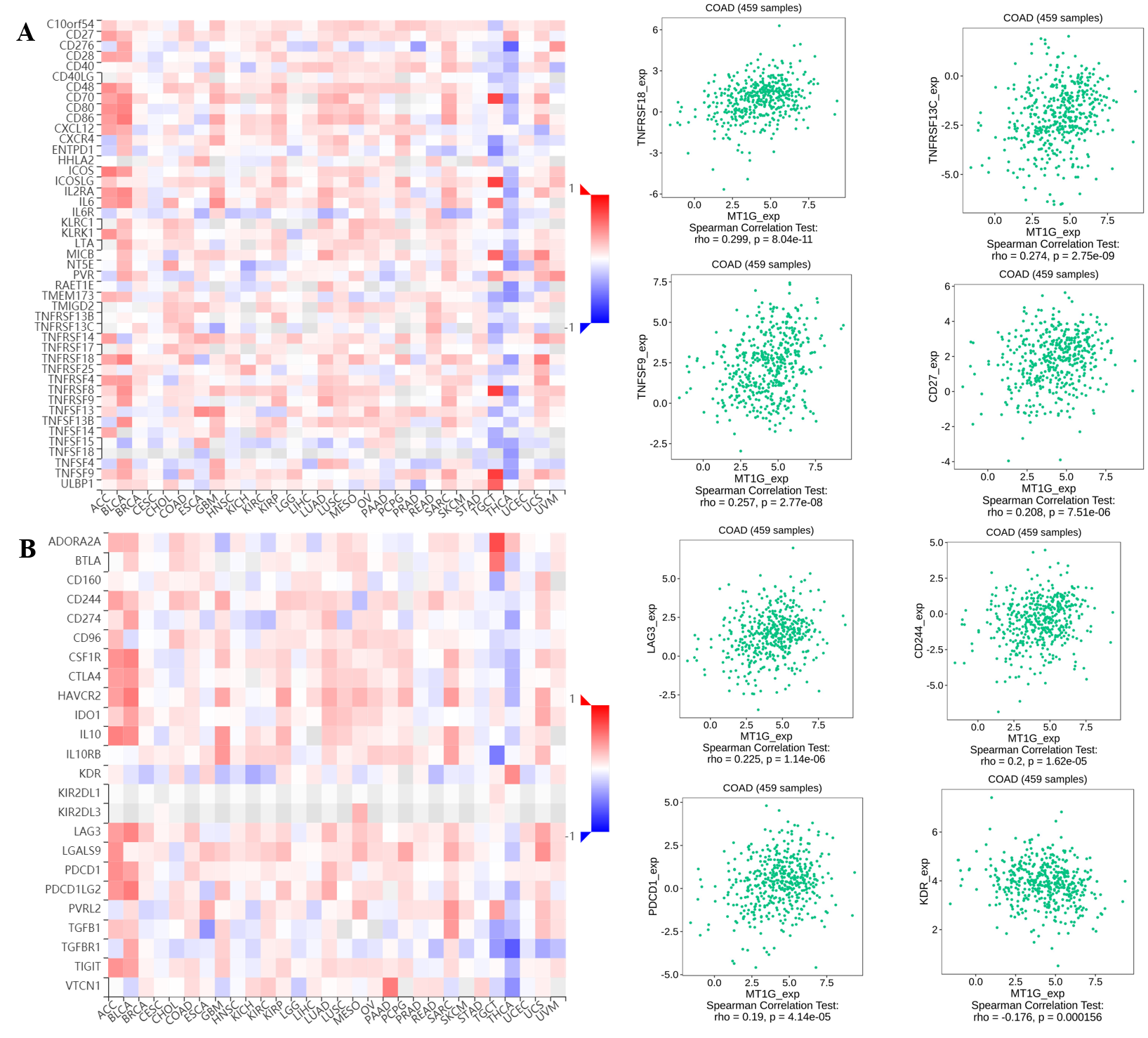

Supplement: Supplementary file 3 [file Image1.TIF]
